# The better to eat you with: morphological disparity and enamel ultrastructure in odontocetes

**DOI:** 10.1038/s41598-023-44112-8

**Published:** 2023-10-08

**Authors:** Mariana Viglino, Martín D. Ezcurra, R. Ewan Fordyce, Carolina Loch

**Affiliations:** 1 Instituto Patagónico de Geología y Paleontología (IPGP), CCT CONICET-CENPAT, U9120ACD Puerto Madryn, Chubut Argentina; 2 Sección Paleontología de Vertebrados, CONICET-Museo Argentino de Ciencias Naturales “Bernardino Rivadavia”, C1405DJR Ciudad Autónoma de Buenos Aires, Argentina; 3 Department of Geology, University of Otago, Dunedin, 9054 New Zealand; 4 Sir John Walsh Research Institute, Faculty of Dentistry, University of Otago, Dunedin, 9054 New Zealand

**Keywords:** Palaeontology, Evolution, Zoology, Palaeoecology

## Abstract

Variations in the shape and size of teeth have been associated with changes in enamel ultrastructure across odontocetes. Characterizing these features in extinct taxa can elucidate their functional morphology and feeding strategy, while also shedding light into macroevolutionary patterns during the evolutionary history of cetaceans. This study aimed to (1) describe the enamel and dentine ultrastructure of the Early Miocene odontocetes *Notocetus vanbenedeni* and *Phoberodon arctirostris* from Patagonia (Argentina) and (2) quantify tooth and enamel ultrastructure morphological disparity among odontocetes. Enamel was predominantly prismatic, thin in the anterior tooth of *N. vanbenedeni* and *P. arctirostris*; whilst thick on the posterior tooth of *N. vanbenedeni*. Together with skull morphology, data suggests a raptorial feeding strategy for *P. arctirostris* and a combination suction feeding method for *N. vanbenedeni*. Statistical analyses supported these inferences, indicating that enamel characters are useful for paleoecological research. Morphological disparity analyses showed that extant odontocetes occupy a larger morphospace and have more disparate morphologies, whilst extinct odontocetes were more similar among each other than with the extant group. There was no clear phylogenetic-based grouping, suggesting that tooth and enamel ultrastructure disparity were mainly driven by ecological pressures. These results highlight enamel ultrastructure as a source for broader-scale paleoecological studies in cetaceans.

## Introduction

During the evolutionary history of cetaceans, their feeding apparatus and teeth underwent substantial morphological changes (e.g.,^1^). Early cetaceans (Archaeoceti) were heterodont and diphyodont, whereas early toothed dolphins (Odontoceti) were heterodont and polydont with comparatively smaller teeth^2^. In early cetaceans, anterior teeth were conical and single-rooted, grading posteriorly to teeth with triangular crowns, accessory denticles, and double roots (e.g.,^2–4^). Modern odontocetes are homodont, with conical single-rooted teeth, while some species are completely toothless (e.g.,^5–8^). Recently described extinct odontocetes from early-diverging lineages also lacked teeth (e.g.,^9,10^). The high degree of morphological variation in teeth from early-diverging to modern odontocetes might correlate to changes in feeding strategies (e.g.,^1,5,9–12^). Most extant odontocetes are either suction-feeders, raptorial predators, or adopt a combination of both behaviors^11,13,14^. Osteological and soft-tissue characteristics are linked to these feeding strategies^5,15,16^, although their relationship is not always straight forward or fully understood. Thus, the functional morphology of teeth can help us elucidate the putative feeding ecology of extinct species.

Due to the high mineral content of enamel and dentine, teeth are among the best-preserved structures in the vertebrate fossil record^17^. Throughout cetacean evolution, morphological variation in tooth shape and size was also associated with changes in enamel ultrastructure. Previous studies^4^ characterized the enamel ultrastructure of some archaeocetes, as well as fossil platanistoids and delphinids, and suggested that the observed enamel simplification reflected possible functional shifts in food processing between these groups. Others^18^ showed that the highly diverse enamel microstructure of extant Odontoceti does not always associate with tooth function or phylogeny. In early archaeocetes, the enamel organization resembles those of extinct ungulates^19^. Another study^20^ analyzed the dental morphology and enamel ultrastructure in one archaeocete and one extinct mysticete, indicating both species as raptorial feeders. Also, previous studies^18^ suggested that complex enamel with Hunter-Schreger Bands (HSB) in *Inia* and *Platanista* is a plesiomorphic condition because HSB are present in archaeocetes and closely-related artiodactyls. These authors suggested that HSB presumably evolved as a response to strong occlusal forces in these early-diverging lineages. Other marine mammals such as fossil and living pinnipeds also have a complex enamel ultrastructure with HSB, although their diet and food processing is relatively similar to that of living cetaceans^8^.

Understanding enamel ultrastructure in extinct odontocetes might help to characterize their functional morphology and putative feeding strategies, contributing to elucidate their paleoecology. There is consensus that ecological factors are linked with macroevolutionary patterns. Cetaceans experienced three radiation events across time^21^. Of those, the second radiation (late Oligocene–Early Miocene) witnessed the origin of Odontoceti and Mysticeti (crown Cetacea). The fossil record indicates a short and rapid diversification of these clades^21,22^ concomitant with the opening of the Southern Ocean, which significantly impacted ocean ecosystems and productivity, among other global climatic events (e.g.,^22,23^). Extinct odontocetes from the second radiation hold important clues to understand a highly significant event in cetacean evolutionary history. In the Chubut Province of Patagonia (Argentina), the Early Miocene fossil record of odontocetes includes at least five species from the Gaiman Formation^24^: *Notocetus vanbenedeni*, *Prosqualodon australis*, *Phoberodon arctirostris*, *Dolgopolis kinchikafiforo*, and *Aondelphis talen*. Despite recent descriptions or revision of these species, including phylogenetic analyses, details on their dental morphology and enamel ultrastructure are poorly known. It is worth noting that among odontocetes from the Gaiman Formation, *Dolgopolis kinchikafiforo* has been described as edentulous^10^ and no teeth are known for *Aondelphis talen*^25^.

This paper aimed to describe the enamel and dentine ultrastructure of the fossil odontocetes *Notocetus vanbenedeni* and *Phoberodon arctirostris* from the Early Miocene of Patagonia. Considering what we know for the enamel ultrastructure of Patagonian *Prosqualodon australis* and other extinct and extant odontocetes, this paper also quantified for the first time their morphological disparity in terms of enamel ultrastructure and dental morphology. We tested for associations with diet, body mass, feeding strategies and habitat, with the aim of characterizing paleoecological adaptations during the second radiation in the evolutionary history of odontocetes.

## Results

### Macromorphology

**Figure 1:**
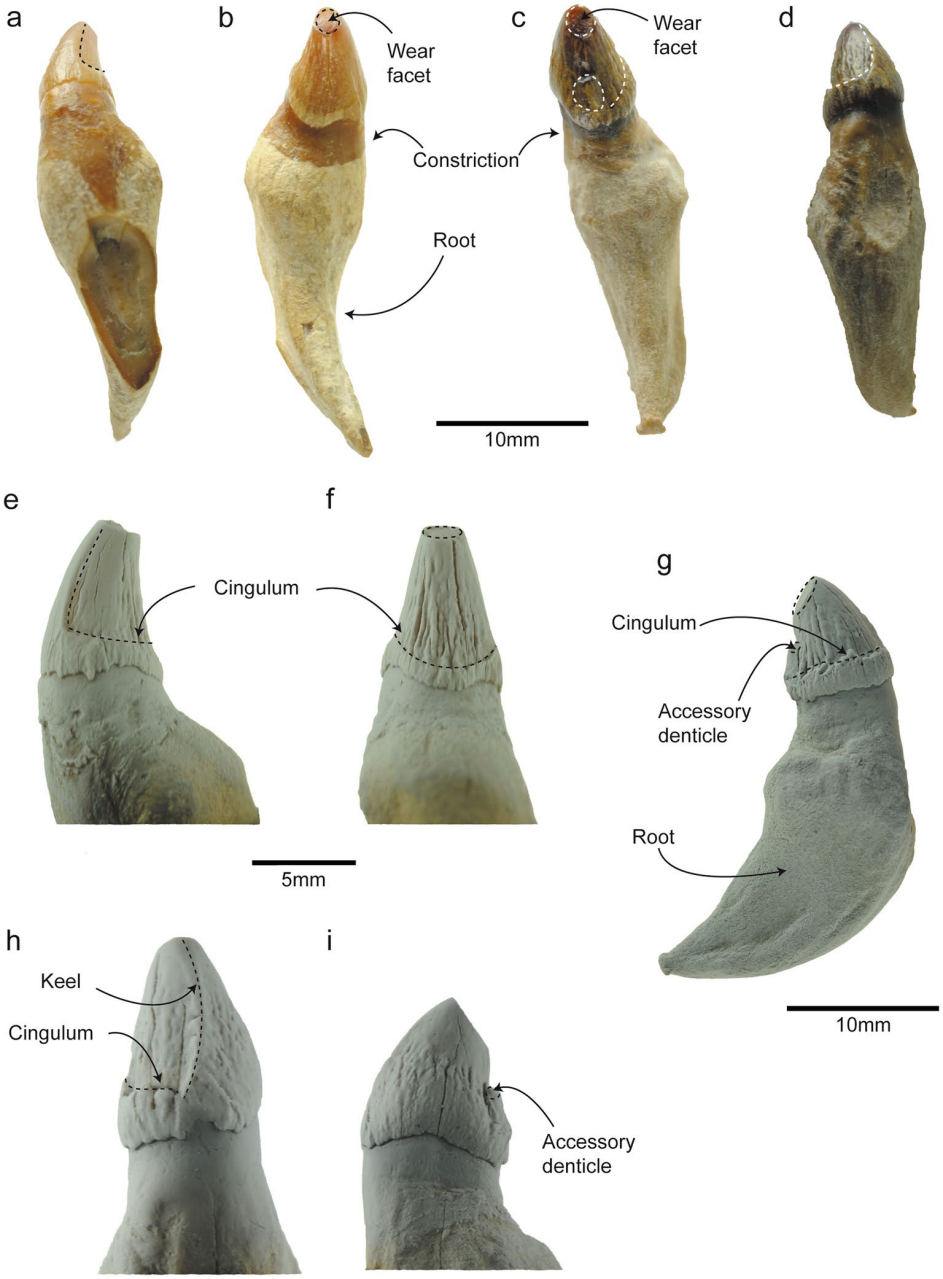
General overview of the anterior and posterior teeth of *Notocetus vanbenedeni* (MPEF-PV 1120). Anterior tooth: (**a**), labial view; (**b**), (**f**), lingual view; (**e**), posterior view. Posterior tooth: (**c**), posterior view; (**d**), antero-lingual view; (**g**), lingual view; (**h**), anterior view; (**i**), labial view. Note that (**e–i**) are coated images.

MPEF-PV 1120 includes an anterior and a posterior tooth of *Notocetus vanbenedeni*, and their identification is based on dental morphological characteristics of the species^26^. The first tooth (Fig. 1a–b,e–f; Table 1) has a conical crown covered by rugose/nodular enamel, with a single cusp showing a cingulum (Fig. 1e, f) without accessory denticles. A weak constriction exposed above the gum line indicates the transition between the crown and the root of this tooth. The root is long and curved bucco-lingually, narrowing towards the apex. These characteristics match the morphology for the most anterior teeth of *Notocetus vanbenedeni*^26^. The tip of the crown has a superficial flat wear facet with distinct smooth edges (Fig. 1b, f), interpreted as an attrition wear facet^14^.

**Table 1:** Measurements (in mm) of the teeth analyzed, following^54^.

	*Notocetus vanbenedeni* (MPEF-PV 1120) Anterior tooth	*Notocetus vanbenedeni* (MPEF-PV 1120) Posterior tooth	*Phoberodon arctirostris* (MPEF-PV 10883) Anterior tooth
Crown length	4.77	7.29	11.71
Crown height	5.40	5.37	13.08
Crown width	7.43	7.68	35.87
Maximum length of tooth	24.09	28.47	64.03 +
Maximum width of tooth	7.26	10.79	13.30

+  = maximum measurement as preserved.

The second tooth (Fig. 1c–d,g–i; Table 1) has a bucco-lingually compressed conical crown with vertically striated enamel. The cingulum at the base of the crown is more marked on the lingual side, being higher posteriorly (Fig. 1d,g). The crown has a worn accessory denticle on the posterior margin and a distinct keel on the anterior margin (Fig. 1g–h). A distinct constriction signals the transition between the crown and the root, exposed above the gum line. The wide, short root is posteriorly curved, narrowing towards the apex. All these characteristics coincide with the most-posterior teeth of other *Notocetus vanbenedeni* specimens^26^. The tip of the main cusp has a polished wear facet with distinct microwear features (Fig. 1c), interpreted as an abrasion wear facet^14^. The constriction zone in both *Notocetus vanbenedeni* teeth could represent glossowear facets^14^, but the presence and direction of surface scratches could not be assessed.

**Figure 2:**
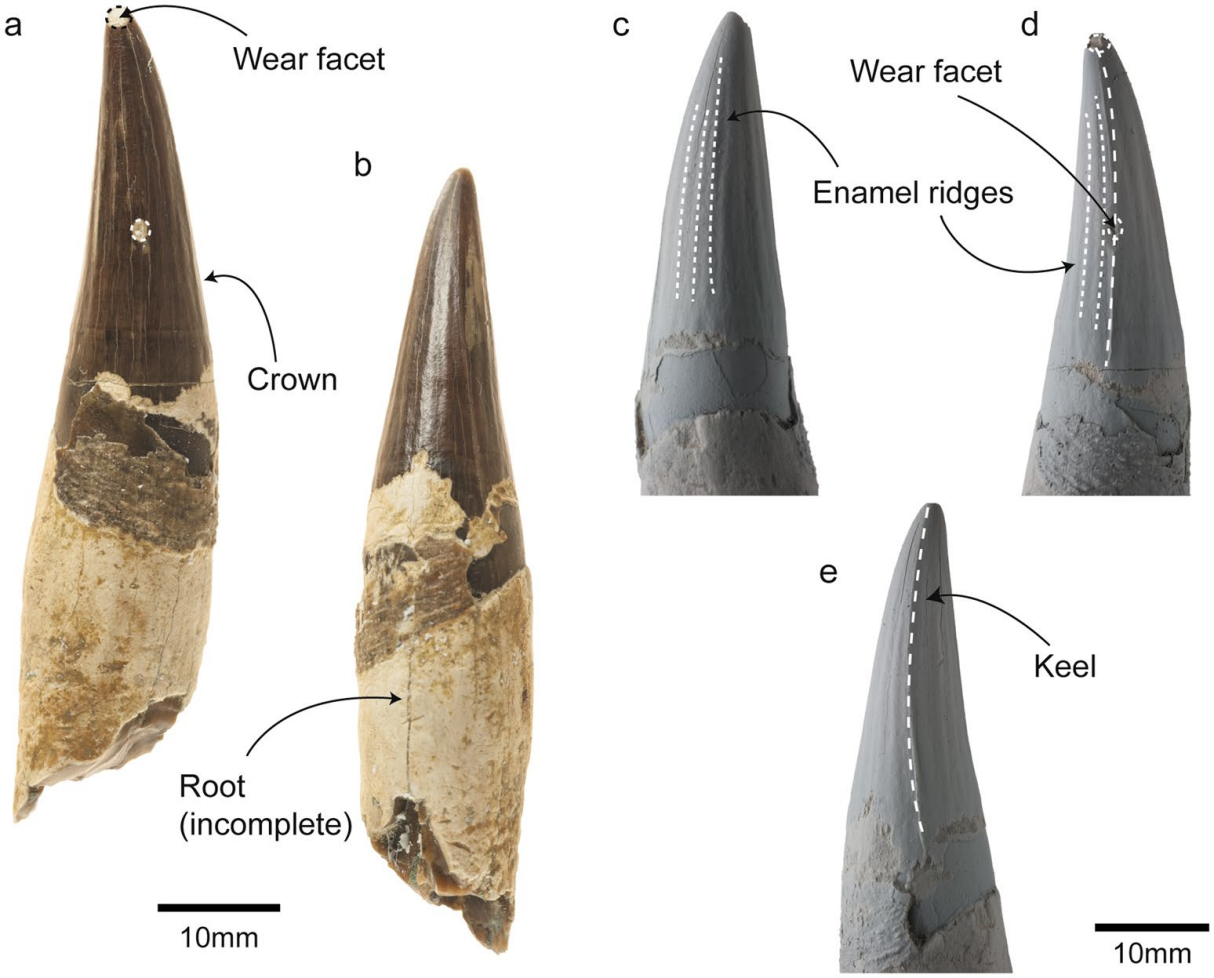
General overview of the anterior tooth of *Phoberodon arctirostris* (MPEF-PV 10883). (**a**), Posterior view; (**b**), Antero-labial view; (**c**), Labial view; (**d**), Posterior view; (**e**), Anterior view. Note that (**c–e**) are coated images.

The tooth of *Phoberodon arctirostris* analyzed here (MPEF-PV 10883) has a conical and elongated crown (Fig. 2a–b; Table 1), anteriorly oriented, with no cingulum or accessory denticles. The enamel has low, vertically-oriented, and subparallel ridges well-separated from each other throughout the crown (Fig. 2c–d) and there is a keel in the anterior surface of the crown (Fig. 2e). Given the general morphology of this tooth in comparison to other preserved teeth for this specimen, it was inferred as a left I1^27^. We observed a marked wear facet on the anterior margin, at approximately half-length of the crown (Fig. 2a). The tip of the crown also has a flat worn facet with smooth edges (Fig. 2a,d). Since both surfaces are flat, without defined edges, they were interpreted as abrasion wear facets^14^.

### Enamel ultrastructure

#### Notocetus vanbenedeni* anterior tooth*

**Figure 3:**
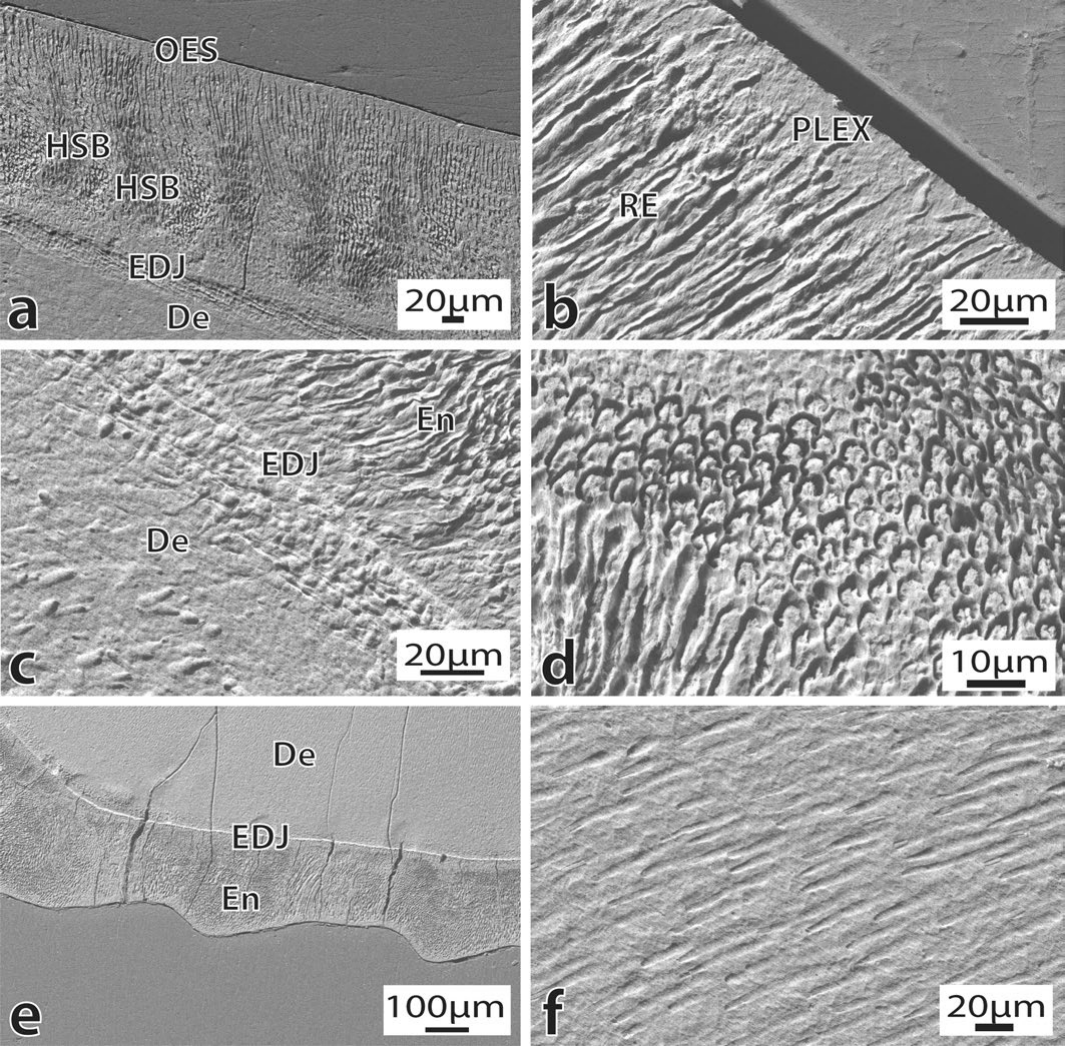
Enamel and dentine ultrastructure of the anterior tooth of *Notocetus vanbenedeni* (MPEF-PV 1120). (**a**), Overview of the enamel in longitudinal section. Magnification 515X; (**b**), Detail of the prismless layer overlaying radial enamel. Magnification 1830X; (**c**), Detail of the flat and sharp EDJ, and underlaying mantle dentine. Magnification 1750X; (**d**), Detail of open enamel prisms. Magnification 2980X; (**e**), Vertical enamel striations seen in cross section. Magnification 235X; (**f**), Overview of the enamel surface showing dentine tubules. Magnification 1060X. Abbreviations: De = Dentine; EDJ = Enamel-dentine Junction; En = Enamel; HSB = Hunter-Schreger bands; PLEX = Prismless enamel; OES = Outer enamel surface; RE = Radial enamel.

The well-preserved enamel of the anterior tooth of MPEF-PV 1120 is prismatic and moderately thin (ranging from 250–270 μm lingually and 270–280 μm bucally). The overall *Schmelzmuster* consists of undulating HSB with narrow transition zones that extend from the enamel-dentine junction (EDJ) towards 2/3 of the enamel layer (Fig. 3a). HSB are 8–10 prisms thick on average and show a slightly inclined undulating course both in cross and longitudinal sections. The outer 1/3 of enamel consists of radial enamel (RE), which fades into a band of prismless enamel 10–20 μm thick, towards the outer enamel surface (Fig. 3b). The EDJ is flat and well-defined (Fig. 3c). The enamel is composed of predominantly open prisms (Fig. 3d), measuring around 3–4 μm in diameter. The interprismatic matrix (IPM) does not seem prominent and is oriented parallel to slightly inclined (10°–20°) to the prism long axes. In cross-section, the vertical striations on the outer enamel surface are evident (Fig. 3e). The dentine shows a smooth surface with dentine tubules scattered throughout the mineralised dentine matrix (Fig. 3f). Biomechanical reinforcing structures resembling tufts and lamellae are present.

### Notocetus vanbenedeni *posterior tooth*

**Figure 4:**
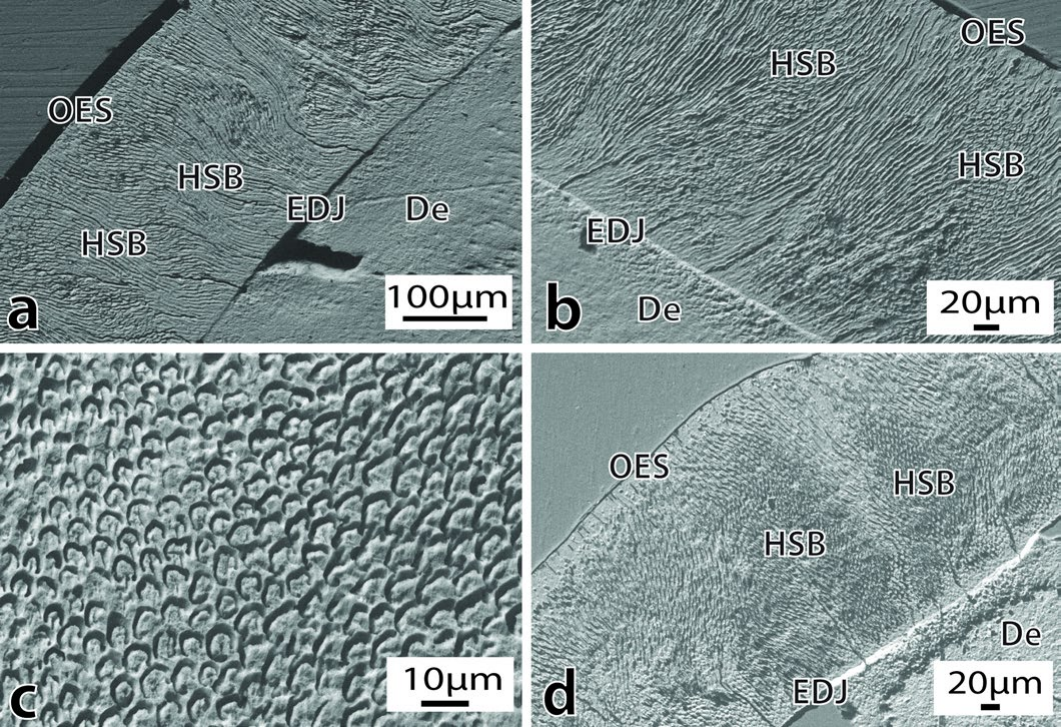
Enamel and dentine ultrastructure in the posterior tooth of *Notocetus vanbenedeni* (MPEF-PV 1120). (**a**), Overview of the enamel ultrastructure in longitudinal view. Magnification 462X; (**b**), Overview of the enamel ultrastructure in cross sectional view, showing evidence of the flat EDJ. Magnification 590X; (**c**), Detail of open enamel prisms. Magnification 2650X; (**d**), Enamel ultrastructure in longitudinal view, with prismless enamel near the OES. Note the diagenetically altered dentine below the EDJ. Magnification 540X. Abbreviations: De = Dentine; EDJ = Enamel-dentine Junction; En = Enamel; HSB = Hunter-Schreger bands; OES = Outer enamel surface.

The enamel in the posterior tooth of MPEF-PV 1120 is also prismatic and moderately thin (280–315 μm lingually and 260–275 μm bucally) (Fig. 4a). The enamel is composed of predominately open prisms (Fig. 4c) arranged in undulating HSB (Fig. 4a,b,d). The HSB extends from the EDJ to the outer enamel surface (OES), where it fades into a band of prismless (PLEX) enamel 10–20 μm thick, which is more evident in cross sections (Fig. 4d). The EDJ is flat and well-defined, without evidence of scalloping (Fig. 4a,b). Biomechanical reinforcing structures resembling tufts and lamellae are present. There is evidence of diagenetic alteration in the mantle dentine below the EDJ (Fig. 4d).

#### Phoberodon arctirostris *anterior tooth*

**Figure 5:**
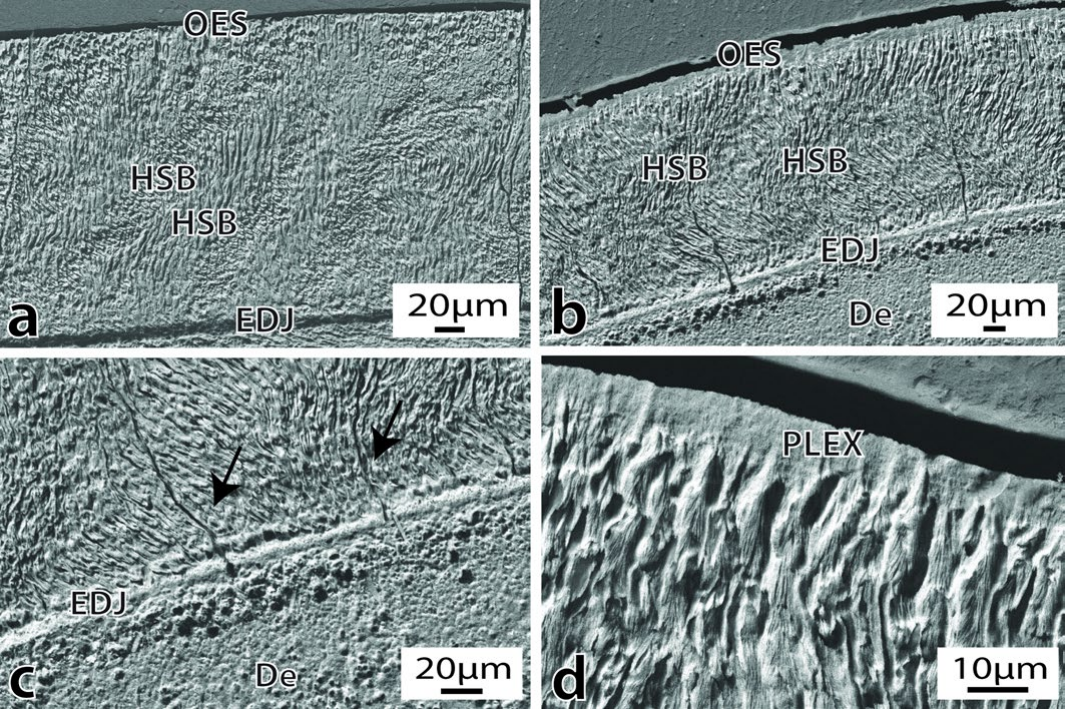
Enamel and dentine ultrastructure in the anterior tooth of *Phoberodon arctirostris* (MPEF-PV 10883). (**a**), Overview of the enamel ultrastructure in longitudinal view. Magnification 560X; (**b**), Overview of the enamel ultrastructure in cross sectional view. Magnification 560X; (**c**), Detail of the flat EDJ, with enamel tufts present (arrows). Note the diagenetic alteration of dentine below the EDJ. Magnification 1220X; (**d**), Detail of the prismless layer near the OES in cross sectional view. Magnification 2980X. Abbreviations: De = Dentine; EDJ = Enamel-dentine Junction; En = Enamel; HSB = Hunter-Schreger bands; PLEX = Prismless enamel; OES = Outer enamel surface.

The prismatic enamel of MPEF-PV 10,883 is composed of mostly open prisms arranged in slightly inclined transversely oriented HSB (Fig. 5a). The IPM is oriented parallel to slightly inclined (10°–20°) to the long axis of the prisms. The enamel is moderately thin (235–265 μm lingually and 210–285 μm bucally, similar to the enamel thickness in the anterior tooth of *Notocetus vanbenedeni*; see above). HSB are 6–8 prisms thick. HSB with narrow transition zones extend from the well-defined flat EDJ to the OES (Fig. 5a, b), where it fades into a band of PLEX enamel 10–20 μm thick, more evident in cross sections (Fig. 5d). There is evidence of diagenetic alteration of the mantle dentine below the EDJ (Fig. 5b,c). Biomechanical reinforcing structures resembling tufts are seen near the EDJ; lamellae-like structures are also present.

### Morphological disparity analyses

**Figure 6:**
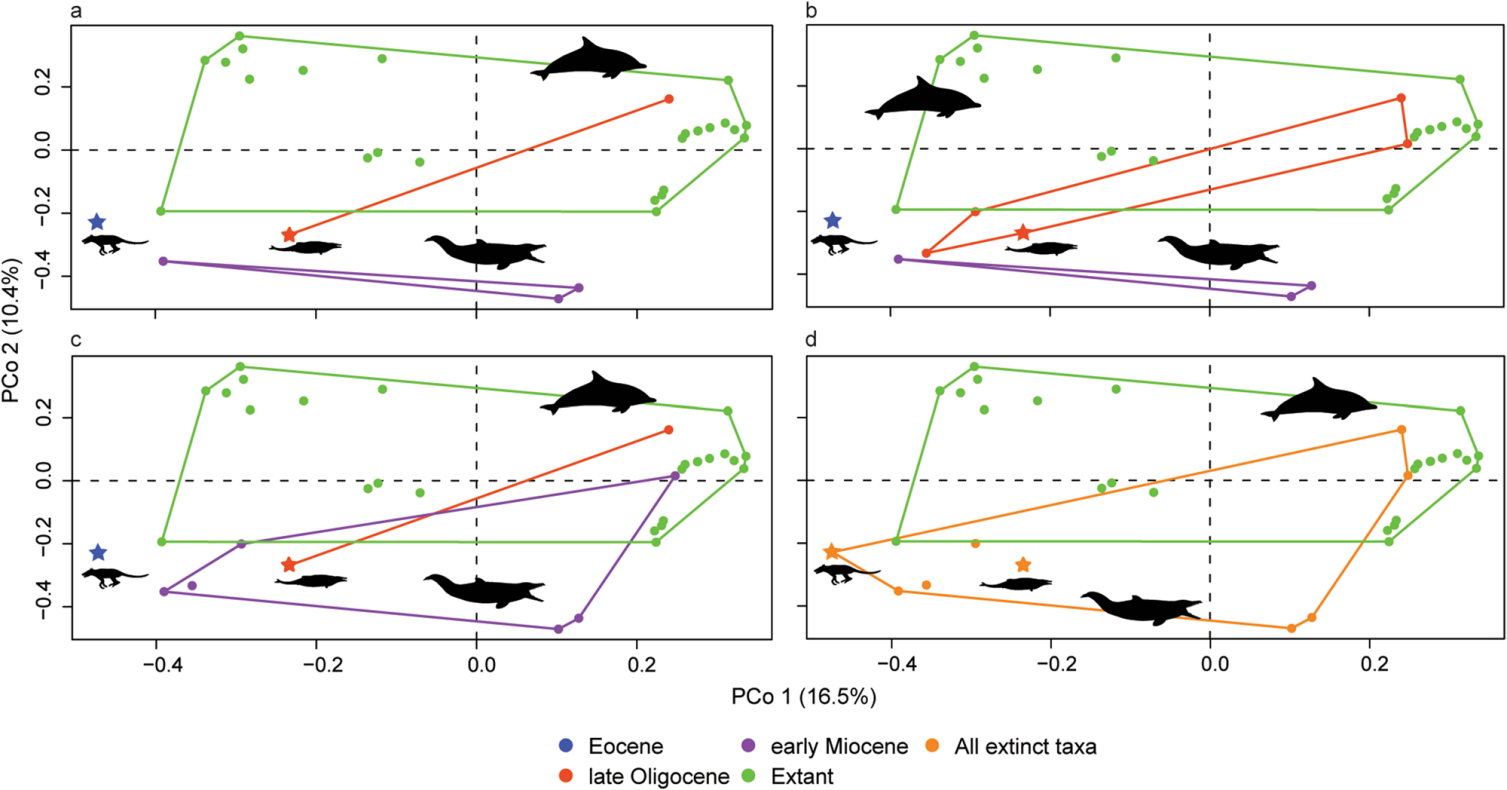
Morphospace in the first two PCo showing the morphological disparity of enamel ultrastructure. Note that extant odontocetes occupy a larger and denser morphospace, which is congruent with the calculated disparity metrics. Stars indicate outgroup terminals. (**a**), “allexcluded” scenario; (**b**), “earlyMioWo” scenario; (**c**), “OligoWo” scenario; (**d**), “fossilsvextant” scenario. Silhouettes indicate each main group (source: PhyloPic).

PCo1–3 account for an accumulated variance of 33.6%. The PCoA shows that extant odontocetes occupy a larger morphospace that partially overlaps with extinct species (except *Pakicetus*) in all four scenarios (*i.e.*, “OligoWo”, “earlyMioWo”, “allexcluded”, and “fossilsvextant”) (Fig. 6). As expected, *Pakicetus* is clearly separated from Odontoceti. It is interesting to note that Late Oligocene kekenodontid OU 22023 is closely plotted with other co-eval extinct odontocetes from our sample. This outgroup specimen is currently under study and awaiting a detailed anatomical description, thus it will be excluded from further discussion. Among extant species, the so-called “river dolphins” (i.e., *Platanista gangetica*, *Inia geoffrensis*, and *Pontoporia blainvillei*) are widely separated from each other in the morphospace, whereas ziphioids and physeteroids plot close to each other in the negative region of PCo1 and the positive region of PCo2 (Figs. 6, ﻿7). All delphinids plot in the positive region of PCo1, clearly separated from the remaining extant odontocetes. Delphinoid OU 22108 is positioned close to its extant representatives, similar to the platanistoid *O. huata*. Phocoenids are scattered across the morphospace without clear separation between clades.

**Figure 7:**
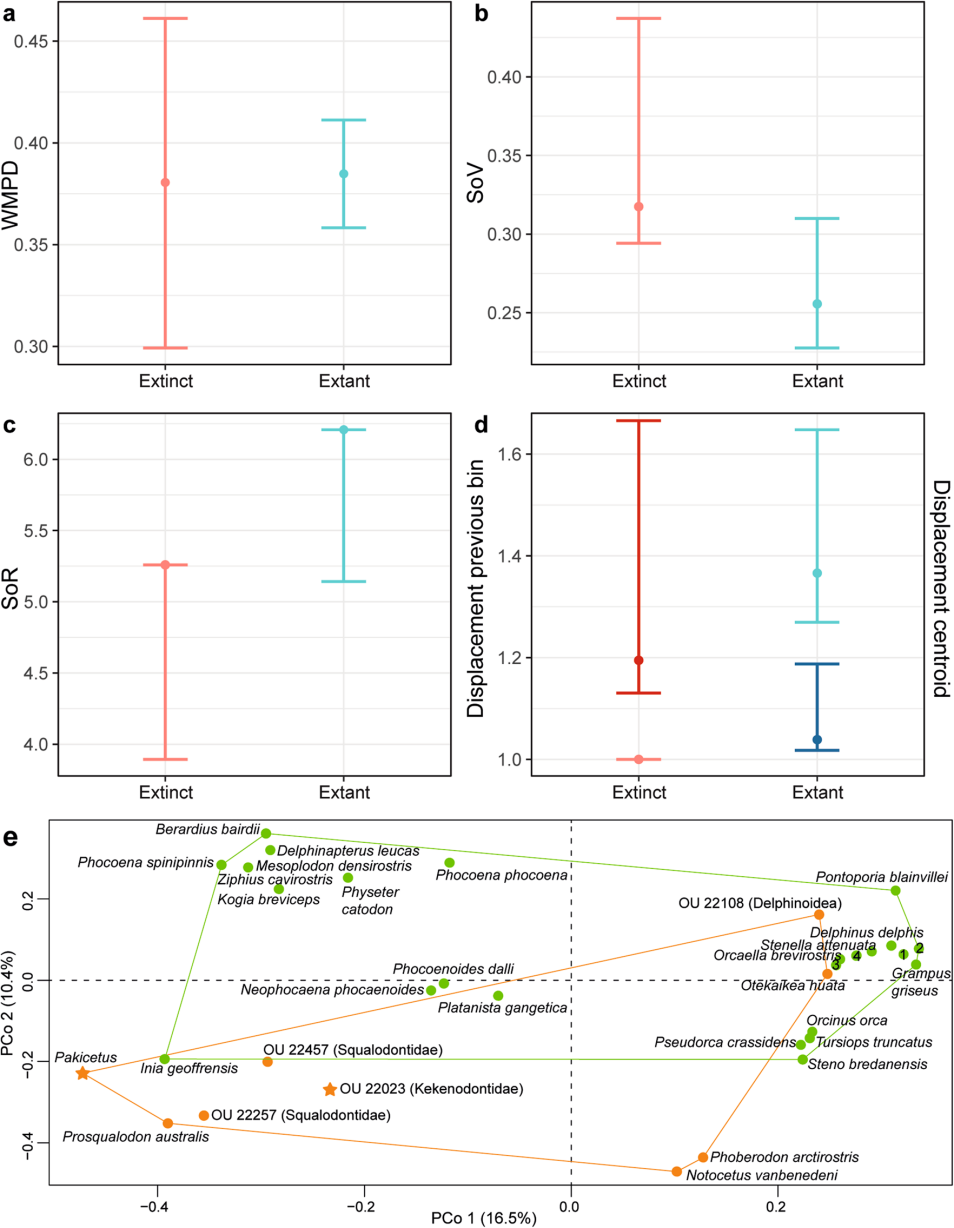
Morphological disparity results based on enamel ultrastructure and dental morphological characters of extinct and extant odontocetes under the “fossilsvextant” scenario. (**a**), WMPD; (**b**), SoV; (**c**), SoR; (**d**), displacement from previous time bin (lighter colours) and displacement from the centroid of the morphospace (darker colours); (**e**), first two PCo axes, indicating the position in the morphospace of all extinct (orange) and extant (green) species included in the present study (for further details of the scenario and metrics, see main text). Stars indicate outgroup terminals. Note the considerable overlap between both groups. References: 1, *Cephalorhynchus hectori*; 2, *Lagenodelphis hosei*; 3, *Globicephala macrorhynchus*; 4, *Lagenorhynchus acutus*, and *Sotalia fluviatilis*.

Disparity metrics show that under the “allexcluded” scenario, Early Miocene taxa occupy a smaller morphospace hypervolume and are more scattered compared to extant taxa; both time bins are also significantly displaced from each other (Fig. 7; Supplementary Table S3 online). Under the “earlyMioWo” scenario, late Oligocene species occupy a larger and denser region of the morphospace than the Early Miocene taxa, but a smaller hypervolume than extant species. Both late Oligocene and Early Miocene time bins have a significantly different position with respect to extant species (Supplementary Table S4 online). Finally, under the “OligoWo” and “fossilsvextant” scenarios, extant species occupy a larger but more scattered distribution in the morphospace than extinct taxa. There are significantly different positions with respect to each time bin in the “fossilsvextant” scenario (Supplementary Table S4 online). Hence, the PCoA shows that extinct odontocetes explored a different region of the morphospace than extant species and the latter group has more disparate dental morphologies. Also, between the late Oligocene and the Early Miocene, morphologies were disparate (i.e., different), although not statistically significant (Fig. 7; Supplementary Table S3 online).

### Categorical variables analyses

In extant species, diet, habitat, and body mass show a significant correlation with morphological disparity of teeth and enamel ultrastructure characteristics (Supplementary Table S5 online).

### Phylogenetic-based analyses

pGLS regressions show that body mass and feeding methods are significantly influencing dental morphological variation and enamel ultrastructure for extinct and extant odontocetes. The interaction of two or all categorical variables also result in statistically significant correlations. However, in all models, the R^2^ was usually low (Supplementary Table S6 online).

**Figure 8:**
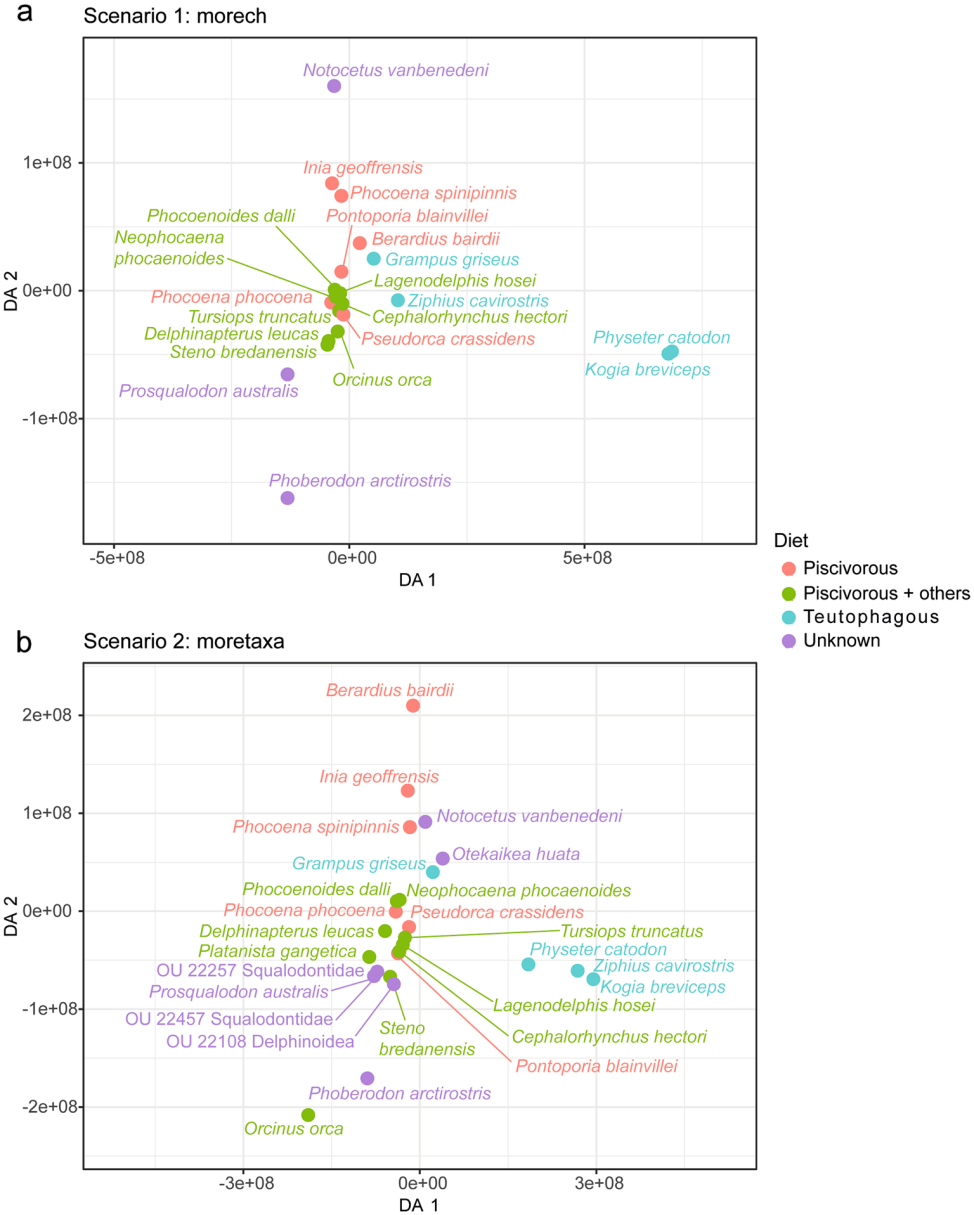
First two DA (Discriminant axes) from the pFDA, showing the different diet categories (see main text for further details). (**a**), “morech” scenario; (**b**), “moretaxa” scenario. Note that in each scenario, extinct species are closely positioned to the extant species with which they share the same diet category.

In both scenarios of the pFDA, the estimated optimal lambda value was 0, suggesting non-significant influence of phylogeny on the discriminant analyses. For the “morech” scenario, the predicted diet for extinct taxa was slightly more accurate (*i.e.*, confusion matrix was better correlated with prior information). The analyses of a wider range of lambda values resulted in predicted diets consistent with previous results (Table 2; Supplementary Table S7 online). Extinct taxa included in both scenarios were assigned in both cases to the same diet. The diet category “Piscivorous_others” was always correctly predicted, while the category “Teutophagus” was almost always correctly predicted. The diet category “Piscivorous” sometimes overlaps with “Piscivorous_others”, but never with “Teutophagus”. In both scenarios, no extinct taxa are predicted as belonging to the diet category “Teutophagus”. Finally, plotting the DA axes in each scenario showed that the predicted diet of extinct taxa is consistent with their position in the discriminant space with respect to that of extant ones (Fig. 8).

**Table 2:** Results of pFDA analyses for both scenarios, with diet inferences for some extinct terminals included in this study, using the optimal and range λ values.

	morech		moretaxa	
	Optimal λ	λ = 0–1	Optimal λ	λ = 0–1
OU 22457 (Squalodontidae)	–	–	Piscivorous_others	Piscivorous_others
OU 22257 (Squalodontidae)	–	–	Piscivorous_others	Piscivorous_others
*Phoberodon arctirostris*	Piscivorous_others	Piscivorous_others	Piscivorous_others	Piscivorous_others
*Prosqualodon australis*	Piscivorous_others	Piscivorous_others	Piscivorous_others	Piscivorous_others
*Notocetus vanbenedeni*	Piscivorous	Piscivorous	Piscivorous	Piscivorous
*Otekaikea huata*	–	–	Piscivorous	Piscivorous & Teutophagus
OU 22108 (Delphinoidea)	–	–	Piscivorous_others	Piscivorous_others

## Discussion

We describe here for the first time the enamel ultrastructure of the Patagonian Early Miocene odontocetes *Phoberodon arctirostris* and *Notocetus vanbenedeni*. The enamel was thin in the anterior tooth of *Notocetus vanbenedeni* and incisor of *Phoberodon arctirostris*, whilst thick on the posterior tooth of *Notocetus vanbenedeni*. The *Schmelzmuster* (i.e., the enamel overall organization) consisted mainly of HSB + RE or HSB + PLEX, being predominantly prismatic. Statistical analyses showed disparate enamel ultrastructure and dental macromorphology characteristics between extinct and extant odontocetes. We could not identify an ecological variable that could explain such variability, indicating that the paleoecological evolution of enamel in odontocetes is more complex than expected. Nonetheless, we highlight the usefulness of enamel ultrastructure information in illuminating paleoecological inferences for extinct taxa and to elucidate the evolution of feeding adaptations in cetaceans.

Enamel in *Notocetus vanbenedeni* was rugose/nodular in texture in both the anterior and posterior tooth, more prominently at the base of the crown; while in *Phoberodon arctirostris* the enamel was smooth with subparallel vertical ridges. Similar enamel ornamentation has also been described for Squalodontidae and *Prosqualodon australis*^4^. Enamel surface ornamentation in many fossil cetaceans has been interpreted as helpful in prey gripping and seizing; however, the functional and phylogenetic significance of enamel ornamentation is still unknown^4^. The enamel of most modern odontocetes is smooth; while surface rugosities can be seen in *Inia geoffrensis*, *Platanista gangetica*, and in the delphinoid *Steno bredanensis*^28^.

The enamel of *Notocetus vanbenedeni* and *Phoberodon arctirostris* is moderately thin, in the 210–315 μm range. These values are slightly lower than average enamel thickness values reported for Squalodontids and *Prosqualodon australis* (350–380 μm)^4^. Enamel thickness in *Notocetus vanbenedeni* and *Phoberodon arctirostris* resembles that of modern odontocetes such as bottlenose and rough-toothed dolphins^28^. As in most fossil and modern cetaceans, the enamel of *Notocetus vanbenedeni* and *Phoberodon arctirostris* is prismatic, composed of mostly open prisms although closed prisms are also observed. Prismatic enamel has been described in archaeocetes^19,20,29,30^, mysticetes^20^, and fossil and modern delphinoids and platanistoids^4,28,31^. Completely prismless enamel has only been observed in some modern phocoenids^28,31^.

The *Schmelzmuster* of *Notocetus vanbenedeni* and *Phoberodon arctirostris* was double-layered and consisted of either of HSB + RE (*Notocetus vanbenedeni* anterior tooth) or HSB + PLEX (*Notocetus vanbenedeni* posterior tooth and *Phoberodon arctirostris* tooth). Undulating HSB is plesiomorphic in cetaceans and has been described in archaeocetes^19,20,29,30^, mysticetes^20^, squalodontids, and *Prosqualodon australis*^4^. In modern cetaceans, HSB is only found in *Platanista gangetica*^30^ and *Inia geoffrensis*^28^. Moreover, the thickness of bands in *Phoberodon arctirostris* (6–8 prisms) was similar to fossil archaeocetes and mysticetes^20^; however, *Notocetus vanbenedeni* had slightly thicker HSB (8–10 prisms). Other marine mammals such as polar bears also present undulating HSB enamel, related to a biomechanically demanding diet^32^. In agreement with previous studies^4^, Early Miocene Patagonian odontocetes show a plesiomorphic *Schmelzmuster*. The enamel of marine *Notocetus vanbenedeni* is similar to the extant representative of its clade (*Platanista gangetica*), suggesting that the evolutionary history leading to riverine habits might not have affected the overall organization of enamel. However, the derived RE + PLEX enamel pattern was observed in the late Oligocene platanistoid *Otekaikea huata*^4^, suggesting that a wider taxonomic survey of enamel ultrastructure data in Platanistoidea could further elucidate the observed evolutionary patterns.

The overall tooth morphology and presence of HSB and biomechanical reinforcement structures such as enamel tufts in *Notocetus vanbenedeni* and *Phoberodon arctirostris* suggest prominent occlusal loads during feeding, consistent with a raptorial feeding strategy. However, facultative suction-assisted raptorial feeding, as common in many long snouted living odontocetes, cannot be discarded. The presence of potential glossowear facets in both *Notocetus vanbenedeni* teeth would bolster the interpretation of suction during feeding for this species^14^. Together with the overall skull and tooth morphology (e.g., shape and length of rostrum, heterodont vs. homodont dentition), a raptorial feeding strategy seems more likely for *Phoberodon arctirostris*, whilst a combination suction feeding method has been proposed for *Notocetus vanbenedeni*^10,26,27^. Late Oligocene squalodontids OU 22457 and OU 22257 and the Early Miocene odontocete *Prosqualodon australis* have been suggested as raptorial feeders, whilst the late Oligocene platanistoid *Otekaikea huata* and delphinoidea OU 22108 have been suggested to perform a combination suction behavior (^4^; but different than^10^ for *Otekaikea huata*). Furthermore, pFDA predicted diet for all these extinct species agreed with their feeding-methods inferences (Table 2; Supplementary Table S7 online). In parallel with feeding methods inferred from skull morphology, these results reinforce that the late Oligocene–Early Miocene was a period of high ecological diversity for odontocetes, particularly among platanistoids^10^.

PCoA analyses and its disparity descriptor metrics showed that extinct odontocetes from the late Oligocene and Early Miocene explored a different region of the morphospace than extant species, with statistically significant differences among them (Figs. 7, 8). Dental morphology and enamel ultrastructure were also different between late Oligocene and Early Miocene terminals, but more similar between each other than with the extant group. In agreement with the general skull and postcranial anatomy (e.g.,^33,34^), *Pakicetus* is clearly separated from odontocetes in the morphospace, clearly influenced by its enamel with more artiodactyl-like characteristics^19^. Contrary to what we expected, PCoA did not recover a clear phylogenetic-based grouping of the terminals in the morphospace, suggesting that ecological pressures rather than common ancestry shaped tooth and enamel disparity during the evolutionary history of odontocetes.

Disparity metrics also showed that extant odontocetes are significantly more diverse in tooth morphology and enamel ultrastructure (Fig. 7). However, when testing ecological variables, body size and feeding methods only slightly influenced enamel ultrastructure characteristics (Supplementary Table S5 online), contrary to what we would have expected. Still, we did not find a single clear ecological characteristic that shaped enamel ultrastructure disparity in odontocetes. Additionally, pGLS analyses yielded significant correlations when two or more ecological variables were considered together (Supplementary Table S6 online). The third radiation (Late Miocene) that gave origin to the modern fauna^21^, opened new ecological niches to be filled. This is supported in our study by the extant group occupying a larger morphospace (Figs. 7, 8). Hence, we hypothesize that complex (rather than single) interactions between environmental and ecological pressures molded a morphological adaptation pathway during cetacean enamel evolution.

We observed an interesting pattern among riverine odontocetes and (their lack of) convergent patterns. The so-called clade “river dolphins” (*Platanista gangetica*, *Pontoporia blainvillei*, *Inia geoffrensis*, and *Lipotes vexiliffer*) include dolphins inhabiting freshwater environments (e.g.,^35^). More recent phylogenetic studies showed “river dolphins” are not part of a monophyletic group, and species have been further allocated in their own families (see^36^ and references therein). The three “river dolphin” species included in our study were distantly located with respect to each other in the morphospace (Figs. 7, 8). Two delphinids included here that also inhabit freshwater environments, *Orcaella brevirostris* and *Neophocaena phocaenoides*^37,38^, are located far apart in the morphospace (Figs. 7, 8). Unlike other skull (e.g.,^39^) and earbones characteristics (^40,41^ but see also^10^), which appear to be convergent among “river dolphin” lineages, enamel ultrastructure seems to have followed a distinct evolutionary path. These results corroborate recent studies showing a weak correlation between enamel ultrastructure and phylogenetic and functional analyses^18^.

Among riverine odontocetes, we would also like to highlight the case of clade Platanistoidea. Extinct species from this group included here (*Notocetus vanbenedeni* and *Otekaikea huata*) are also distantly positioned from each other and from their extant representative *Platanista gangetica* in the morphospace (Figs. 7, 8). This is not surprising, given their disparate enamel characteristics (Supplementary Tables S3 and S4 online). For example, *Otekaikea huata* has a *Schmelzmuster* of RE + PLEX^4^, whilst *Notocetus vanbenedeni* has either HSB + RE or HSB + PLEX (Figs. 4, 5). *Platanista gangetica*, on the other hand, has a predominant HSB pattern^30^. Future studies increasing the taxonomic sampling and investigating enamel ultrastructure could shed light on when feeding adaptations related to riverine habitats were acquired in platanistoids.

The late Oligocene–Neogene has been suggested as a period of burst in tooth morphologies and ecological niches for marine mammals^42,43^. It is not surprising that the second radiation event in the evolutionary history of cetaceans occurred around this time^21^. A strong ecological pressure during the evolutionary history of marine tetrapods shaped the unique polydont homodont dentition we see today in odontocetes^15^, with many characteristics already acquired as early as the late Oligocene–Early Miocene. The analyses presented here show, for the first time, a quantified disparate variation in enamel ultrastructure among odontocetes. Despite the small number of terminals used (and the associated long error ranges), we were able to confidently recover a clear disparate pattern along the evolutionary history of odontocetes.

The evolution and phylogenetic significance of enamel features shows a complex and unclear pattern. Despite providing clues to elucidate feeding biomechanics and function, the taxonomic, phylogenetic, and functional significance of many enamel features are still debatable, as they might be likely influenced by convergence and homoplasy^4,18,44^. Further studies on enamel ultrastructure data as presented here, together with skull morphology and isotope analyses (e.g.,^45^), could provide more robust paleoecological inferences for extinct odontocetes.

## Methods

### Materials examined

Fossil teeth analyzed here are from two odontocetes from the Gaiman Formation: *Notocetus vanbenedeni* and *Phoberodon arctirostris* (Early Miocene, Burdigalian;^46^). Here, we analyzed two detached teeth, one anterior and one posterior tooth, belonging to *Notocetus vanbenedeni* (MPEF-PV 1120). Both teeth were identified by comparisons with other *Notocetus vanbenedeni* specimens with teeth in situ. The specimen was identified to species-level as it was associated with taxonomically-diagnostic skull and tympanic bulla^26^. We also analyzed a detached tooth belonging to *Phoberodon arctirostris* (MPEF-PV 10883). It was identified as an incisor due to its conical crown and long root, in comparison to teeth from the same specimen still attached to the skull. The species-level identification was possible because the tooth was associated with a taxonomically-diagnostic skull^27^.

### Sample preparation for enamel ultrastructure

Silicone molds of the studied teeth were made, and epoxy resin replicas produced, to record the original 3-dimensional structure before destructive sampling; replicas are held in the Colección de Paleontología de Vertebrados of the Museo Paleontológico Egidio Feruglio (MPEF-PV), Trelew, Argentina. Sample preparation followed^4,28^. Teeth were surface-cleaned with alcohol and embedded in epoxy resin (Epofix Cold-Setting Embedding Resin, Struers, Copenhagen, Denmark) using silicon molds. After setting for 24 h, specimens were sectioned using a MOD13 diamond wheel in an Accutom-50 high-speed saw (Struers, Copenhagen, Denmark) under water-irrigation. Cross-sectional and longitudinal sections were polished in a TegraPol-21 polisher (Struers, Copenhagen, Denmark) with silicon carbide paper (1200, 2400, and 4000 grit) and sonicated in water for 3 min after polishing with each grit size. Final polishing was achieved with 1 μm diamond paste (Struers, Copenhagen, Denmark). Specimens were sonicated in ethanol for 1 min for final removal of any residual debris. To reveal detailed enamel structure, polished fossil teeth were etched with 2 M hydrochloric acid for up to 8 s, following^47^). Teeth were then sonicated in water for 1 min. Polished and etched samples were surface coated with gold–palladium for scanning electron microscopy (SEM) observation. Secondary electron microscope images were obtained in a Zeiss Sigma VP FEG-SEM housed at the Otago Micro and Nanoscale Imaging facility (OMNI), Dunedin, New Zealand. The SEM operated at 15 kV and magnification ranged from 75 to 3000X.

### Morphological disparity analyses

Morphological analyses were based on a morphological matrix built here for this purpose, which is composed of 11 characters (ch) and 35 species-level and specimen-level terminals (Supplementary Data S1 and S2 online). Characters were defined to best capture enamel ultrastructure and dental morphological variation. The novel enamel ultrastructure information described here for the Patagonian odontocetes *Notocetus vanbenedeni* and *Phoberodon arctirostris* was included in this data matrix. Terminals included both extinct and extant Odontoceti specimens/species, including all currently recognized extant families^18^. The outgroup comprises the archaeocete genus *Pakicetus* and the kekenodontid specimen OU 22023 (Geology Museum, University of Otago, Dunedin, New Zealand)^4,19^. We also built a semi-strict phylogenetic supertree based on previous phylogenetic hypotheses^26,36,48^.

Based on the chronostratigraphic uncertainty or range of the species (Supplementary Table S1 online), we defined four time bins: (i) Eocene (56–33.9 Ma); (ii) Oligocene (33.9–23.03 Ma); (iii) Early Miocene (23.03–15.97 Ma); and (iv) Late Miocene–Present (15.97–0 Ma). Given that three taxa overlap in two time bins (*Otekaikea huata*, OU 22257, and OU 22457), we estimated all pre- and post-ordination disparity metrics under four different scenarios: excluding the overlapped taxa from the younger time bin (“earlyMioWo”); excluding the overlapped taxa from the older time bin (“OligoWo”); excluding all overlapped taxa (“allexcluded”); and separating all taxa into extinct and extant groups (“fossilsvextant”).

Morphological disparity was quantified using the package Claddis v. 0.6.3^49^ for R environment^50^ and the matrix described above. A distance matrix was generated using maximum observable rescaled distances (MORD), including a hierarchy matrix that aids in differentiating missing data from inapplicable characters^51^. The pre-ordination weighted mean pairwise dissimilarity (WMPD) disparity metric was calculated based on the obtained distance matrix.

We performed a Principal Coordinates Analysis (PCoA) based on the distance matrix; the PCoA included a Lingoes correction due to the presence of negative eigenvalues. For subsequent analyses, we used the first 12 coordinates (61.5% accumulated variance), which were selected visually by identifying the last major change of slope in the scree plot of percentages of explained variance for each coordinate. Post-ordination disparity metrics were calculated in order to describe the multidimensional morphospace using the R package dispRity v. 1.6.0^52^: Sum of Variances (SoV), Sum of Ranges (SoR), functional divergence, mean displacement from the centroid, and mean displacement from the centroid of the previous time bin. We applied the test.metric function of the dispRity package to measure how these metrics capture the size, density, and position of the morphospace. SoV and SoR successfully capture density and size but not position. Functional divergence successfully captures size, density, and position; and the mean displacements accurately captured morphospace positions. Statistical significance between groups was assessed through the non-overlap of 95% confidence intervals calculated from 9,999 bootstrap replicates (boot.matrix() function of dispRity) of the dissimilarity matrix for WMPD and the ordinated matrix for the post-ordination disparity metrics, and the subsequent recalculation of the measures. The 95% confidence intervals were generated using the two tails (0.025%) of the population of values recovered from the resampled matrix. This procedure was followed for all the disparity metrics except for SoR, because in SoR the resampled values cannot be higher than the original value of the non-resampled dataset. As a result, we used only one tail (0.05%) of the population of resampled values to build the 95% confidence intervals in SoR. We also calculated the above post-ordination metrics using rarefied matrices to the minimum number of taxa sampled on a time bin to test how sensitive these metrics were to sample differences. Statistical significance between groups was assessed as detailed before.

The position in the morphospace between time bins was statistically tested using a permutational multivariate analysis of variance (PERMANOVA) analysis (adonis2 function of the R package vegan v. 2.5–7^53^) with 9999 permutations and the Euclidean method.

### Categorical variables explanatory analyses

We conducted an exploratory analysis of the relationship between enamel ultrastructure, dental morphological characters, and different paleoecological variables to test whether they could explain the observed morphological variation. The categorical variables defined were as follows: (i) habitat (semi-aquatic, freshwater, coastal marine, oceanic marine, and polar); (ii) diet (freshwater prey, piscivorous, piscivorous and others, teutophagous, and top predator); (iii) body mass (maximum value in kg regardless of whether it was a male or female; for extinct species, we estimated their body mass following^54^ or^55^); and (iv) feeding method (semi-aquatic feeding, raptorial, combination suction, and capture suction; based on^10,11,13^) (see further details in Supplementary Table S2 online).

We built a distance matrix using the vegdist function of the R package vegan based on the categorical variables outlined above. We then conducted a PCoA including the Lingoes correction as before. We tested for a statistically significant relationship by fitting a linear model between PCo scores and each of the categorical variables.

### Phylogenetic-based analyses

We conducted phylogenetic generalized least square (pGLS) regressions to test the association between categorical variables (see above) and the first three PCos initially obtained. We applied the procD.pgls function of the R packages geomorph v. 4.0.4 and RRPP v. 1.3.0^56–59^ using the type II sum of squares error and 999 iterations. These analyses allowed for phylogenetic dependence between species to be accounted for.

We conducted a phylogenetic flexible discriminant analysis (pFDA) in order to maximize between-groups discrimination, including a phylogenetic covariance component. pFDA considers the phylogenetic bias when predicting a categorical factor based on quantitative variables. The goal was to analyze whether specific enamel ultrastructure and dental morphological characters could maximize the differentiation between ecological groups of odontocetes according to their diet, and predict diet for the analyzed extinct species. We followed^60^ and^61^ analyses for the R environment. We tested two scenarios, based on the exclusion of terminals and characters with missing data: (1) we chose to preserve more characters than extinct terminals (“morech”) (ch = 10, taxa = 22); or (2) we chose to maintain as many extinct terminals as possible (“moretaxa”) (ch = 7, taxa = 26). In both scenarios, terminals with inapplicable characters and/or polymorphisms were given a new state number, as the analyses do not allow these scorings. Also, the character “Accessory denticles” was deleted, as the coded state for all extant taxa was the same. To increase the statistical power of the analyses, *Orcinus orca* was re-categorized as “Piscivorous_others”. We estimated the optimal lambda value and the predicted diet for extinct taxa, as well as predicted their diet across a wide range of lambda values (i.e. 0–1 every 0.1 intervals). We then plotted the first two discriminant axes (DA).

### Supplementary Information

Supplementary Information 1.

Supplementary Information 2.

Supplementary Information 3.

## Data Availability

All data matrices and R scripts are available in the Supplementary data files online.
